# Therapeutic Targeting of Cancer Stem Cells in Lung, Head and Neck, and Bladder Cancers

**DOI:** 10.3390/cancers13205098

**Published:** 2021-10-12

**Authors:** Sarah E. Mudra, Pritam Sadhukhan, M. Talha Ugurlu, Shorna Alam, Mohammad O. Hoque

**Affiliations:** 1 School of Medicine, University of Louisville, Louisville, KY 40202, USA; sarah.mudra@louisville.edu; 2 Department of Otolaryngology, Head and Neck Surgery, Johns Hopkins University School of Medicine, Baltimore, MD 21231, USA; psadhuk1@jhmi.edu (P.S.); mugurlu1@jhmi.edu (M.T.U.); shorna@mit.edu (S.A.); 3 Department of Oncology, Johns Hopkins University School of Medicine, Baltimore, MD 21231, USA; 4 Department of Urology, Johns Hopkins University School of Medicine, Baltimore, MD 21231, USA

**Keywords:** cancer stem cells, cancer recurrence, therapeutic resistance, tumor microenvironment, signaling pathways, targeted therapy, head and neck cancer, lung cancer, bladder cancer

## Abstract

**Simple Summary:**

Effective cancer treatment hinges upon overcoming therapeutic resistance mechanisms that allow for the continued proliferation of cancer cell subpopulations. Exposure to pharmacotherapy invariably leads to resistance as tumor cells with selected advantageous features evade destruction and alter the tumor composition. Cancer stem cells (CSCs) with features of plasticity that allow for regeneration and differentiation are particularly responsible for this phenomenon. Advances in tumor biology and molecular signaling have highlighted their role in neoplastic initiation, invasion, and maintenance. Novel strategies to direct therapy against these tumor cell subpopulations have the potential to dramatically alter tumor response and change the course of cancer care.

**Abstract:**

Resistance to cancer therapy remains a significant obstacle in treating patients with various solid malignancies. Exposure to current chemotherapeutics and targeted agents invariably leads to therapy resistance, heralding the need for novel agents. Cancer stem cells (CSCs)—a subpopulation of tumor cells with capacities for self-renewal and multi-lineage differentiation—represent a pool of therapeutically resistant cells. CSCs often share physical and molecular characteristics with the stem cell population of the human body. It remains challenging to selectively target CSCs in therapeutically resistant tumors. The generation of CSCs and induction of therapeutic resistance can be attributed to several deregulated critical growth regulatory signaling pathways such as WNT/β-catenin, Notch, Hippo, and Hedgehog. Beyond growth regulatory pathways, CSCs also change the tumor microenvironment and resist endogenous immune attack. Thus, CSCs can interfere with each stage of carcinogenesis from malignant transformation to the onset of metastasis to tumor recurrence. A thorough review of novel targeted agents to act against CSCs is fundamental for advancing cancer treatment in the setting of both intrinsic and acquired resistance.

## 1. Introduction 

**Figure 1:**
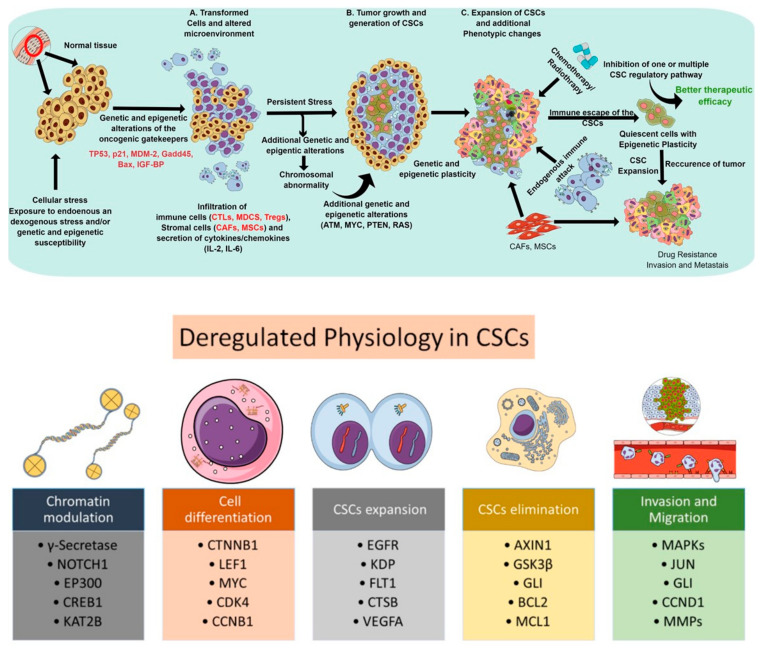
Schematic representations depicting the deregulated physiological process and subsequent steps involved in the proliferation, expansion, elimination, and functionality of cancer stem cells. The upper panel represents the progression of CSC development to drug-resistance and metastasis. The bottom panel highlights some key molecules involved in enabling CSCs to expand, escape first-line therapy, evade immune response and, ultimately, progress to widespread metastatic disease.

Cancer stem cells (CSCs)—a subpopulation of slow-growing, tumor-initiating, self-renewing cells within solid tumors—are associated with tumorigenesis, progression, chemoresistance, and recurrence. In light of cancer-associated pathophysiology, these cells bear the capacity for self-renewal and are long-lived in the tumor microenvironment [1]. Various studies over the past decades have established CSCs as one of the key players behind cancer recurrence via tumor dormancy, neoangiogenesis, immune evasion, and metastasis. Although several theories about the lineage of CSCs exist, the most widely adopted theory suggests CSCs differentiate from hematopoietic stem cells (HSCs) and maintain a quiescent (or dormant) state. Other work has proposed that CSCs have distinct cellular characteristics which make them very abundant and highly proliferative. CSCs are regarded as the prototype of normal stem cells, as they exhibit similar cell surface receptors (e.g., CD44, CD24, and CD133), molecular pathways (e.g., Wnt/β-catenin, Notch, and Hedgehog), and phenotypes (e.g., self-renewal and differentiation), but behave in a deregulated manner. CSCs display altered cell cycle kinetics, DNA replication and repair mechanisms, as well as express antiapoptotic and transport proteins [2]. They facilitate tumor initiation, maintenance, heterogeneity, invasiveness, and therapeutic resistance. [3,4,5]. Thus, a dedicated study of the molecular mechanisms responsible for cell cycle regulation, self-renewal, DNA repair, and cellular senescence among normal stem cell lines remains foundational. The ability to distinguish CSCs in the tumor microenvironment may subsequently lead to the development of directed therapies against CSC-regulating pathways. In principle, targeting CSC-regulating pathways may allow for the eradication of tumor-initiating cells and augment current systemic therapies which simply reach rapidly dividing cell types. Thus, it is critical to uncover the interplay between CSC-regulating pathways in the setting of intrinsic and acquired therapeutic resistance (Figure 1). Figure 1 gives an overview of the process by which normal tissue becomes carcinogenic. It also details key markers and mechanisms that facilitate drug resistance, immune evasion and the persistence of CSCs. 

**Figure 2:**
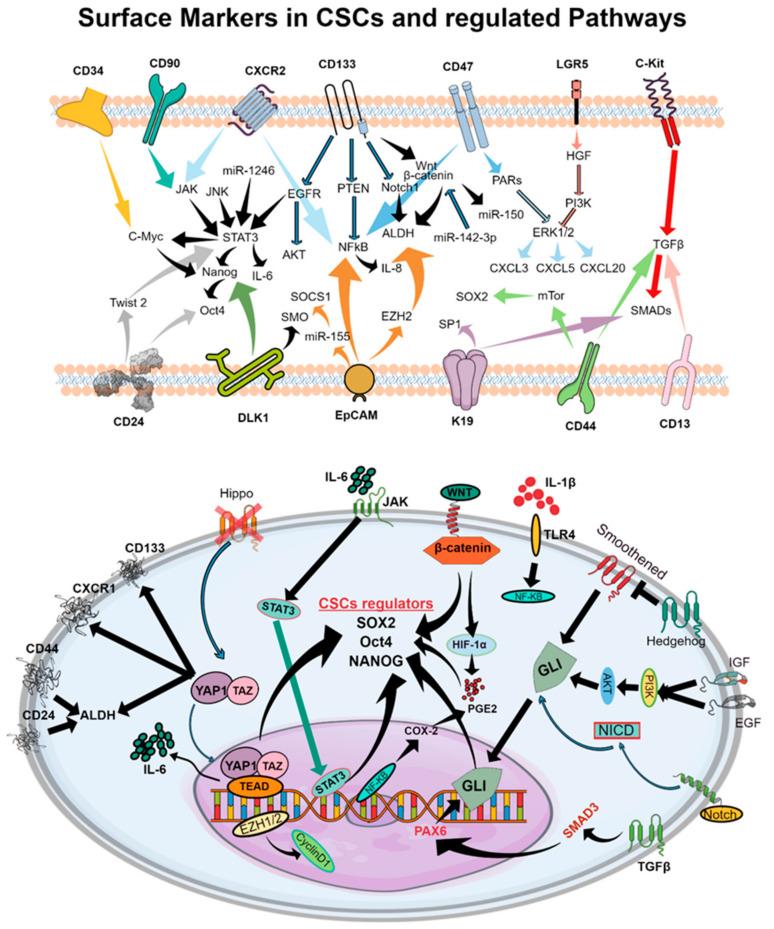
Schematic representations of cell surface molecules and downstream signaling pathways involved in CSC function which facilitate tumor progression and the development of therapeutic resistance. The upper panel represents cell surface markers of CSCs which regulate signaling cascades that maintain the CSC phenotype in the tumor microenvironment. In the bottom panel, pathways leading to the production of SOX2, Oct4, and NANOG, the principal regulators of the CSC phenotype, are shown in detail. Cell surface markers and subsequent intracellular intermediates which govern cell proliferation and maintenance of the CSC phenotype are highlighted.

Despite major advances in therapeutic strategies which induce gene inhibition through antisense oligonucleotides [6], CRISPR/Cas9 [7], ribozymes [8], and long noncoding RNAs critical for immune escape [9], our present knowledge of the complex biology of CSCs limits the use of these molecular techniques to design curative therapies [10]. CSCs govern variable cellular behaviors, treatment responses, and metastatic abilities [3,4]. An interconverted phenotype where cells undergo an epithelial–mesenchymal transition (EMT) and/or mesenchymal–epithelial transition (MET) facilitates the acquisition or enrichment of markers of stemness in CSCs [11]. These EMT and METs are also characterized by the highly quiescent and invasive behavior of CSCs. CSCs have been shown to exhibit characteristic features including cell surface markers (e.g., CD34, CD90, CD133, CD44, CD166, α2β1 integrin, CXCR2, CD47, LGR5, c-Kit) [12] (Figure 2), enzymes (e.g., ALDH1) [13], drug efflux transporters (e.g., P-glycoprotein, ATP-binding cassette proteins) [14], and morphological features (e.g., spheroid formation) [15]. Recent evidence suggests that CSCs may facilitate therapeutic resistance by repopulating residual tumors between chemotherapy cycles [2,16]. However, detailed mechanisms of CSC regulation and effective strategies for overcoming therapeutic resistance remain largely unknown. The presence of cell surface markers may vary between samples from different patients, or even within a patient’s own tumor due to microenvironmental factors including hypoxia, stromal cells, and epigenetic alterations. CSC marker expression even varies between tumor stages or tumor tissue types. Certainly, the future of effective cancer treatment requires a thorough understanding of the CSC-regulating and -expanding pathways crucial for tumor maintenance, drug resistance, and metastasis. 

CSC populations arise following genetic and/or epigenetic alterations of normal stem cells or deregulated progenitor cell signaling that leads to aberrant plasticity. These cells evade chemotherapy, as conventional chemotherapies target highly proliferative cells and CSCs exist in a quiescent state. As a component of the tumor microenvironment, CSCs possess a high degree of heterogeneity with cancer-associated fibroblasts (CAFs), comprising a key component (Figure 1). These highly proliferative CAFs escape chemotherapy via the reprogramming of autophagic pathways [17]. Furthermore, driven by growth factors and cytokines, CSCs display high metabolic activity that renders them resistant to chemotherapy [18,19]. Targeting aberrant antioxidant signaling mechanisms may prove useful in selectively killing CSCs [20,21]. 

Advancements in tumor biology and cancer signaling have underscored the role of CSCs in cancer initiation, progression, invasion, and resistance. Recent research has heralded novel strategies to target CSCs through cell surface markers, molecular pathways, and microenvironmental features. Combining conventional cytotoxic agents with targeted therapies has increased therapeutic efficacy and prolonged survival among patients with various solid malignancies. However, this treatment strategy invariably generates resistant cell subpopulations. Therapeutic resistance remains challenging for cancer management, particularly among patients previously treated with targeted therapies. As targeted agents and immunotherapies emerge and alter the landscape of cancer treatment, their efficacy must be measured against these persistent, tumor-maintaining CSCs. Thus, targeting pathways required for the generation, survival, or function of CSCs will enhance long-term responses to current systemic therapy. Undoubtedly, drugs targeting CSCs could be crucial for overcoming therapeutic resistance and achieving durable curative responses. 

At present, data regarding the impact of FDA-approved targeted agents to eliminate CSCs remains uncertain. Previously, CSCs were thought to display strikingly similar features across cancer subtypes. Recent research, however, has called this into question. Tumors have been found to display significant inter- and intratumor heterogeneity. Even still, consensus is lacking regarding markers distinguishing CSCs. It is well known that pharmacotherapy and/or the tumor microenvironment itself may encourage the transition from non-CSC tumor cells to CSCs [22]. For these reasons, it remains difficult to isolate and interpret cancer-type-specific CSCs. Therefore, in this review, we separately summarize the current research on developing and marketed therapeutics which may directly or indirectly target CSCs in lung, head and neck, and bladder cancers. We also discuss the present evidence regarding the efficacy of these CSC-targeted therapies alone or in combination with conventional therapy.

## 2. CSC Signaling Pathways Implicated in Therapeutic Resistance

Dysregulated and aberrant signaling of molecular pathways—including JAK/STAT, Notch, Hedgehog, WNT, PI3K, PTEN, and NF-kB—have been implicated in CSC generation, expansion, and subsequent tumor generation [13] (Figure 2). In particular, CSC-regulating pathways such as Notch, WNT, Hippo, STAT-3, and Hedgehog have been identified as key contributors to drug resistance [5,19,21,23,24]. 

Aberrant Notch signaling has been well documented in both non-small-cell and small-cell lung cancers [25]. In particular, elevated Notch signaling has been associated with CSC self-renewal, metastasis, and angiogenesis, and may represent a potent target for overcoming therapeutic resistance and metastasis [15]. 

Canonical WNT signaling regulates a myriad of cellular processes throughout embryonic development. However, WNT also plays a role in stem cell renewal [26]. Dysregulated WNT signaling has been implicated in various malignancies including breast, colon, and skin cancers [26]. 

Hedgehog signaling has been shown to influence the renewal and survival of CSCs in various tumors including lung cancer. In a study of lung squamous carcinoma, both in situ and severely dysplastic lung tissues displayed increased levels of nuclear β-catenin compared to normal and metaplastic lung tissues [27]. A study of preinvasive lung squamous-cell carcinoma correlated elevated β-catenin signaling with increased cell proliferation, disease severity, and features of EMT [27]. The necessity of CSC features to induce EMT has been reported [28]. Levina et al. demonstrated a correlation between high nuclear β-catenin levels and lung CSCs, as evidenced by tumor sphere formation and high metastatic potential when transplanted into NOD/SCID mice [29]. Silencing of β-catenin activity via siRNA has shown promise for reducing CSC proliferation and the subsequent development of drug resistance in lung cancer [30]. 

In summary, the inhibition of CSC-generation- and expansion-associated pathways is critical for eliminating CSCs [12] and enhancing the efficacy of systemic therapies. Furthermore, both intrinsic and acquired resistance mechanisms to targeted therapies must be overcome in order to augment treatment with demonstrated utility against CSC-regulating pathways [31]. 

## 3. Unique Properties of CSCs by Cancer Type

### 3.1. Features of CSCs in Lung Cancer 

Globally, lung cancer is the most common cancer caused by both extrinsic and intrinsic factors. Therapeutic resistance and metastasis are critical challenges to curative lung cancer treatment. Specifically, CSCs are the most significant obstacle for overcoming these challenges. Researchers have identified lung CSC markers including CD133, ALDH1A1, ALDH1A3, CXCR4 [32,33], CD44 [34], CD90 [35], CD166 [36], and uPAR [37]. For instance, lung cancer cells expressing CD133 demonstrate increased stemness features, adhesion, motility, and drug efflux ability [14,19,24,38]. NANOG, OCT4, CD133, EpCAM, NCAM, and CEA correlate with high capacity for self-renewal, proliferation, differentiation, and chemoresistance [39]. These markers have also been shown to recapitulate tumor heterogeneity and mimic specific tumor histology in murine xenografts [39]. Although these markers aid in the identification of lung CSCs, their utility as drug targets is challenging considering both intratumoral as well as interpatient heterogeneity. Furthermore, these markers are variably expressed; for example, research regarding CD133 and CD44 as specific markers of lung cancer CSCs is conflicting, making drug targeting increasingly complex [10].

The ATP-binding cassette subfamily B member 1 (ABCB1) has also been associated with CSC properties, the EMT, and acquired resistance to tyrosine kinase inhibitors in NSCLC [40]. Beyond surface markers, transcription factors including OCT4 and BMI1 have been implicated in CSC-defining properties such as self-renewal and invasion [41,42]. In a population of lung cancer CSCs, the knockdown of OCT4 led to apoptosis [41]. Additionally, OCT4-enriched ABCG2 expression also correlated with increased capacity for self-renewal and chemoresistance [42]. In another study, insulin-like growth factor 1 mediated chromatin modifications that resulted in phenotypic CSC heterogeneity [27]. 

The above-noted features of CSCs may play a primary role in the development of therapy resistance—altering tumor biology and impacting patient outcomes [38]. For example, in patients with NSCLC treated with platinum-based chemotherapy, high expression of CD133 was negatively correlated with progression-free survival [38]. Expression of ALDH1 and SOX2 was also correlated with elevated disease stage and grade in lung adenocarcinoma [19].

#### 3.1.1. Therapeutic Targeting of CSCs and CSC-Regulating Pathways in NSCLC

Despite the overall efficacy of EGFR tyrosine kinase inhibitors (TKIs) in lung cancer treatment, most patients develop TKI resistance within 8-10 months [43,44]. For 40% of patients, the resistance mechanism is uncertain [45]. Invariably, EGFR blockade results in therapeutic resistance through enhanced CSC activity [46,47,48,49]. Even prior to treatment, lung cancer CSCs possess markers of stemness, including CD133, OCT4, and Nanog. Increased HES1, BMI1, and ALDH1A expression are correlated with heightened TKI resistance and diminished survival among patients with EGFR-positive NSCLC [48]. 

Knockdown of CSC-regulating pathways involved in cancer stemness may augment sensitivity to current chemotherapy and targeted therapy like EGFR TKIs [50]. A number of TKIs are approved for use in EGFR-mutant lung cancer. Gefitinib and erlotinib are widely used to augment treatment, and are indicated for upfront therapy in patients with EGFR-mutant NSCLC [51]. Potential CSC markers are detailed by cancer type in Table 1 and Table 2. A summary of some CSC pathway targeting drugs is provided in Table 3. 

##### EGFR TKIs

***Gefitinib***

Present research indicates that gefitinib has limited efficacy in eradicating CSC populations. A recent report demonstrated that gefitinib inhibits the transcription factor and stem cell regulator Sal-like protein 4 (SALL4) in EGFR-mutant, CD44-positive NSCLC cell lines [51]. Another group found increased expression of FOXO3a, a forkhead family transcription factor, correlated with poor gefitinib response [45]. They reported that epigenetic alterations of NF-kB through microRNA-155 (miR-155) enhanced features of stemness both in vitro and in vivo, and promoted gefitinib resistance independent of EGFR mutation status [45]. Others reported increased Oct4 and CD133 expression among gefitinib-resistant cells, both in vitro and in vivo [73]. Other lung cancer cell lines resistant to gefitinib showed increased ALDH1A1 expression, EMT features, and self-renewal capacity [74]. Samples from tumors resistant to gefitinib also displayed ALDH1A1 upregulation [74]. Zinc finger E-box-binding homeobox 1 (ZEB1) was also found to be upregulated in gefitinib-resistant EGFR-mutant NSCLC patients; clinical studies showed that ZEB1 maintained CSC traits through the regulation of miR200c and BMI1 in a gefitinib-resistant model [75]. Some studies suggested that the expression of CSC molecules such as CD47 may also facilitate adaptive resistance and immune evasion of cancer cells. Targeted therapy to block CD47 was effective against wild-type and EGFR-mutant lung cancer cells [71].

Therapeutic agents targeting these markers may overcome gefitinib resistance. β-Elemene, a bioactive molecule isolated from a Chinese herb, has been shown to regulate the expression of EZH2, and can also act synergistically with gefitinib in targeting CSC-like traits in aggressive lung cancers [76]. Another study found that all-trans retinoic acid also decreased CSC-mediated resistance in gefitinib-treated lung cancer cells [57].

***Afatinib***

Afatinib was first found to suppress ATP-binding cassette subfamily G member 2 (ABCG2) activity, self-renewal capacity, and tumorigenesis in patient-derived leukemia cells—specifically, via ABCG2 promoter methylation [77]. Thus, ABCG2 has been proposed as a promising biomarker to target CSCs [55]. Although these initial data were observed in leukemia, afatinib exerts similar activity in solid tumor types [77]. Afatinib also inhibits ABCG2 efflux activity and suppresses its expression in human colon, breast, and nasopharyngeal carcinoma cell lines as well as in leukemic bone marrow [77]. The combination of afatinib and topotecan enhanced antitumor activity in vitro and in vivo, supporting dual therapy with both a targeted agent and conventional chemotherapy [77]. Others identified the epigenetic silencing of miR-200c—a suppressor of EMT—and overexpression of CSC markers such as ALDH1A1 and ABCB1 as mediators of afatinib resistance [64]. In EGFR-positive head and neck cancer and lung cancer cell lines, elevated expression of EGFR and PD-L1 levels occur concurrently. Additionally, features of stemness accompanied elevated PD-L1 levels in EGFR-positive cancers. Treatment with afatinib inhibited STAT1 and IFR-1 levels, subsequently repressing PD-L1 expression [62]. Thus, afatinib may possess a dual role in mediating tumor cell death via (1) suppression of CSC features and (2) repression of PD-L1 [66]. 

In both gefitinib-sensitive and gefitinib-resistant EGFR-mutant NSCLC cell lines, afatinib exhibited antitumor activity [48]. Codony-Servat et al. found that afatinib induced phosphorylation of STAT3 (pSTAT3) at tyrosine 705, resulting in elevated pSTAT3 and RANTES expression [48]. Afatinib, in combination with a STAT3 inhibitor, decreased pSTAT3, STAT3, and RANTES mRNA levels [48]. Despite these initially promising data, dual treatment with afatinib and a STAT3 inhibitor failed to eradicate a population of NSCLC CSCs [48]. Following treatment, an ALDH+ cell population emerged [48]. However, this study suggests the promise of novel small molecule inhibitors in combination with afatinib to increase response and prolong progression-free survival in lung cancer [48]. 

***Erlotinib***

Studies have begun to shed light on the utility of erlotinib to eradicate CSCs. However, the literature reveals variable efficacy and cites the involvement of various signaling pathways and resistance mechanisms. 

Lung cancer cells continuously exposed to erlotinib showed enhanced features of stemness, including self-renewal, differentiation, and enhanced expression of OCT3, OCT4, Nanog, Sox2, and ID2 [31]. Unsurprisingly, prolonged erlotinib exposure diminished therapeutic sensitivity [31]. In one study of NSCLC, sphere cells expressing MAP17, CD133, and ABCG2 increased following erlotinib treatment, suggesting a population of erlotinib-resistant CSCs [67]. Upregulation of microRNA-23a (miR-23a) may provide one means to mediate EGFR-TKI resistance by suppressing the PI3K/AKT pathway and subsequently inducing apoptosis [65]. While Han et al. found that erlotinib inhibited EGFR, the drug failed to suppress the phosphorylation of PI3K/AKT in CSCs [65]. Both miR-23a knockdown and miR-23a inhibition enhanced erlotinib sensitivity and re-sensitized CSCs to erlotinib-induced cell death through enhanced PTEN expression [65]. The suppression of another microRNA, miR-223, may also play a role in erlotinib resistance through aberrant IGF1R/PI3K/AKT signaling [63]. Following erlotinib treatment, a subpopulation of CD133-positive cells emerged [63]. These cells possessed CSC features including self-renewal, pluripotency, and tumorigenicity in vitro, and even greater resistance to erlotinib occurred in vivo [63]. Iderzorig et al. demonstrated the roles of p120-catenin, Kaiso factor, and PRMT-1 in EMT activation targeting PRMT-1/p120-catenin-enhanced erlotinib sensitivity [28]. These findings suggest the need to develop novel strategies to target CSCs in order to overcome TKI resistance. Progesterone receptor membrane component 1 (PGRMC1) has been shown to impact drug resistance and cancer stemness in breast, colon, lung, and thyroid tumors [78]. In lung-tumor-derived CSCs, PGRMC1 was overexpressed [78]; while treatment with erlotinib did not trigger cell death in this CSC subpopulation, treatment with a PGRMC1 inhibitor showed efficacy against these CSCs, supporting PGRMC1 as a CSC marker and drug target [78]. 

Little et al. demonstrated that DUOX1 silencing enhanced erlotinib resistance, tumor invasiveness, and expression of CD133 and ALDH1 [61]. This silencing may directly influence EMT, as DUOX1-silenced lung cancer cells displayed increased migration, anchorage-independent growth, and vimentin and collagen expression [61]. Hence, DUOX1 silencing may contribute to erlotinib resistance and failure to eradicate CSCs. MDM2, a negative regulator of p53, failed to translocate from the nucleus following erlotinib treatment [79]. The pharmacological and genetic inhibition of MDM2 restored p53 signaling, reduced features of stemness, and inhibited tumorigenicity [79]. Among lung cancers with aberrant MDM2 signaling, anti-MDM2 agents may enhance CSC eradication. 

A recent study reported that gefitinib, erlotinib, and afatinib each induced STAT3 phosphorylation and enhanced ALDH expression [48]. Following EGFR inhibition, surviving cell subpopulations possessed high ALDH expression and CSC features. Other candidates including STAT3, integrin/focal adhesion kinase, and Src have been shown to maintain stemness in cancer cell populations [48]. In an EGFR-mutant NSCLC cell line, spheroid cells expressing CD133, CD44, Oct4, and ABCG2 were resistant to erlotinib [67]. However, the concurrent inhibition of EGFR, STAT3, and Src increased CSC eradication and therapeutic efficacy [50]. 

Some lung cancer cell lines sensitive to erlotinib express PD-L1 and MHC-I. Overall, erlotinib inhibited PD-L1 expression—another manner in which EGFR inhibitors may augment T-cell-mediated cancer killing [59]. Although these researchers did not directly examine features of stemness, they noted high PD-L1 expression correlated with increased STAT3 expression—a proposed CSC marker [59]. A preclinical study demonstrated that erlotinib is effective in EGFR-WT tumors phosphorylated at tyr1068 [60]. Overall, despite a wealth of published data, the efficacy of erlotinib in inhibiting lung CSCs remains uncertain. Further studies are essential in order to augment the therapeutic efficacy of erlotinib in combination with other agents targeting CSC-generating and -expanding pathway(s). 

***Osimertinib***

Osimertinib, a third generation TKI, has been less widely studied for its efficacy in eradicating CSCs. In EGFR-mutant NSCLC cell lines, treatment with gefitinib or osimertinib activated STAT3 and Src-YAP1 signaling pathways implicated in stemness. Thus, in part, acquired resistance to osimertinib is the result of persistent CSC-like cells [48]. In a lung adenocarcinoma cell line, combination treatment with osimertinib and a STAT3 and/or Src inhibitor decreased ALDH+ cells [50]. Apart from this, upregulation of shisha3 was potentially effective in reducing tumor growth in a TKI-resistant LUAD model [80].

##### Other Small Molecule Inhibitors

***Crizotinib***

The ALK/ROS1 inhibitor crizotinib has primarily been examined in echinoderm microtubule-associated protein-like-4-anaplastic lymphoma kinase (EML4-ALK)-positive NSCLC [81]. The EML4-ALK fusion protein influences downstream molecules including STAT3, ERK, and AKT—each noted for their role in CSC induction and maintenance [82]. Furthermore, increasing concentrations of crizotinib inhibited the expression of long intergenic noncoding RNA regulator of reprogramming (linc-ROR) which plays a role in acquiring and maintaining CSCs as well as chemoresistance in NSCLC [81]. These data suggest that linc-ROR may overcome crizotinib resistance and serve as target in EML4-ALK+ NSCLC [81]. Recently, crizotinib has been studied in combination with rapamycin—an mTOR inhibitor and CSC-targeting agent [81]. Together, crizotinib and rapamycin enhanced cell death and re-sensitized crizotinib-resistant EML4-ALK+ NSCLC cells. This synergy may result from the dual AKT-mTOR pathway inhibition of crizotinib and rapamycin. Treatment with crizotinib alone decreased the downstream expression of NANOG, OCT4, and ALDH. Furthermore, crizotinib-treated cells lost their sphere-forming abilities in a dose-dependent manner, suggesting that crizotinib at least partially inhibits features of stemness in EML4-ALK+ NSCLC cells [81]. In principle, dual therapy with crizotinib and rapamycin may enhance the eradication of CSCs. 

***Imatinib***

Levina et al. discovered that the expression of proto-oncogene receptor tyrosine kinase, c-kit, and the production of stem cell factor (SCF) maintained NSCLC CSCs [29]. Inhibition of c-kit signaling (via imatinib) and SCF (via neutralizing antibodies) decreased CSC proliferation [29]. The combination of imatinib or anti-SCF antibodies with conventional chemotherapy (cisplatin) inhibited both bulk tumor cells and CSC subpopulations, although more research is needed in order to validate this single study’s findings [29].

##### Other Agents with Unknown CSC Activity 

Beyond the drugs outlined above, numerous FDA-approved targeted inhibitors are used in lung cancer treatment. These include inhibitors of ALK (alectinib, ceritinib, lorlatinib), EGFR (necitumumab, dacomitinib), mTOR (afinitor), ROS1 (brigatinib), and PD-L1 (durvalumab) (Table 2). To our knowledge, these agents have yet to be investigated in a CSC-specific context. Further examination of these agents in relation to CSC pathways may yield fruitful opportunities to expand the durability and efficacy of cancer therapy. 

### 3.2. Features of CSCs in Head and Neck Cancers

Head and neck cancers (predominately head and neck squamous-cell carcinoma (HNSCC)) are among the most heterogeneous cancer types. In HNSCC, CSCs comprise approximately 1–5% of the tumor population. Unlike other cancer types, the impact of CSCs in head and neck cancers is widespread, impacting cancer initiation, metastasis, and drug resistance [54]. Treatment with radiation and/or chemotherapy presents a principal mechanism by which tumor cells gain features of stemness. In the heterogeneous tumor microenvironment, CSCs exhibit effective DNA repair mechanisms via increased expression of DNA repair and pro-survival genes. The highly heterogeneous nature of HNSCC impedes the discovery of unique and specific CSC markers. However, markers of stemness such as CD44, ALDH1, CD133, CD166, CD98, BMI1, NANOG, OCT4, and SOX2 have been identified in head and neck cancer cells [54]. In particular, increased CD44 expression in head and neck cancer was associated with increased capacity for tumor initiation and differentiation [53]. Moreover, CD133 was found to be associated with tumorigenesis, cell proliferation, and differentiation in tongue, laryngeal, buccal, and oral cancer [52]. In a different study, CD47 and CD133 were found to be promising as predictors of CSC proliferation. Unlike in lung cancer, CD47 can be considered as a targeted therapeutic agent in HNSCC patients [69]. One group noted high CD117 expression in oral cancer cells; however, more studies are required in order to establish its presence in CSCs of other head and neck cancers. A recent study supports the idea that integrin β1 regulates cellular stemness and integrin β1 signaling correlated with Notch1-mediated expression of Hey1 and Hes1 in HNSCC [72]. Recently, different groups reported that long intervening/intergenic noncoding RNAs (lincRNAs) play a critical role in inducing CSC-like properties in head and neck carcinomas. A preclinical study identified LINC00319 as a key player in regulating the expression of CSC traits in laryngeal squamous-cell carcinoma [83].

#### 3.2.1. Therapy Targeting CSCs in Head and Neck Cancers

##### EGFR TKIs

***Afatinib***

In addition to acting as an EGFR inhibitor, afatinib acted against a CSC-like population of human nasopharyngeal cells, presumably via the suppression of transport activity and downregulation of ABCG2 [77]. In vivo, afatinib impaired tumorigenicity, suppressed tumor growth, and sensitized CSCs to traditional chemotherapeutics. Thus, afatinib in combination with traditional chemotherapy may enhance the eradication of CSCs [77]. Mechanistically, in vitro and in vivo, afatinib decreased levels of STAT1 and IRF-1—JAK/STAT signaling pathway candidates posited as players of cancer stemness [62]. In turn, afatinib reduced STAT1 and IFR-1 expression as well as decreased PD-L1 levels, which may, in turn, enhance the efficacy of immunotherapy [62]. 

***Cetuximab***

Recent findings by one group suggest that cetuximab fails to act upon CSCs expressing CD44, CD133, and CD117. Cetuximab-treated CSCs possess a greater proliferation rate compared to non-CSC subpopulations [56]. However, compared to treatment with paclitaxel, cetuximab-treated cell lines were less resistant [56]. These data suggest that cetuximab should not be used as a single agent, although more studies are required. A second group noted variable spheroid formation among cetuximab-resistant and cetuximab-sensitive head and neck cancer cell lines [84]. While cetuximab-resistant cells continued to form spheroids, this morphology was inhibited in cetuximab-sensitive cells [84]. In sum, these data indicate that features of stemness persist in cetuximab-resistant cells. Targeted therapeutics likely display variable efficacy against heterogeneous tumors with complex microenvironments. Therefore, additional targeting is required in order to eliminate these CSCs and develop precision-driven therapy. 

***Erlotinib***

The study of erlotinib to target head and neck CSCs has yielded unpromising data. In CSC populations, erlotinib diminished cell proliferation, yet demonstrated little efficacy in inducing cell death. This may be due, in part, to tumor heterogeneity. Following three erlotinib treatments, CSC populations were preserved. CSCs remained that expressed increased CD44, decreased epithelial surface antigen (ESA), and mesenchymal markers including vimentin and e-cadherin. No change was noted in sphere-forming capacity [70]. In another study, erlotinib selected for ALDH-expressing CSCs, yet contributed to chemotherapy and radiotherapy resistance in vitro [85]. 

##### Other Agents with Unknown CSC Activity

Several studies have identified novel molecules that may dampen the CSC phenotype in cancer cells. PTC-209 is an inhibitor of Bmi1/AP-1-driven cascade, and has been shown to be synergistically effective with cisplatin [86]. BGJ398 specifically inhibits FGF in HNSCC cells and can effectively target CSC molecules in high-ALDH- and CD44-expressing cells [87]. Additionally, specific inhibitors for ALDH1, Alda 89 and Aldi-6, had synergistic effects in combination with cisplatin to inhibit cell proliferation and CSC features [88]. PF-2341066, an inhibitor of cMET, has also been shown to inhibit tumor-initiating factors and metastatic phenotypes in HNSCC samples [89].

### 3.3. Features of CSCs in Bladder Cancer 

In 2009, researchers first identified CSCs in urothelial carcinoma of the bladder (UCB) [90]. Thereafter, studies have continued to support CSCs as a critical player in chemoresistance and morbidity. At present, CSC markers in UCB include ALDH1, AR (androgen receptor), CD44, ABCG2, COX2/PGE2, YAP1/STAT3, Wnt/β-catenin, P63, BMI, EZH1, EZH2, SOX2, OCT4, SOX4, PARP1, HDACs, and SMO. In UCB patients, CSC markers including CD24, CD47, and CD47 have been used to develop targeted therapy as well as demonstrate high prognostic value among patients who have undergone radical cystectomies [91]. Notably, these markers are exclusively upregulated in CSC populations, and are not characteristic of normal stem cell populations [19,21]. 

Following extensive molecular characterization and study of severity and aggressiveness, UCB is broadly classified into two types: (1) non-muscle-invasive (NMIUCB), and (2) muscle-invasive (MIUCB) [92]. Within these subtypes, stem cell populations and characteristics vary widely. CSCs of NMIUCB display some basal characteristics including CD44, CK5, P-cadherin, and CK14 expression. Other markers such as ALDH, Nestin, CD133, and CD90 identify CSCs of MIUCB. Upregulation of genes including NANOG, OCT4, and SOXs has been associated with aggressive features of MIUCB, including chemoresistance and self-renewal [21,92].

The Wnt/β-catenin pathway is a known regulator of EMT and the phenotype of UCB CSCs. This pathway also contributes to tumor progression by enhancing tumor-initiating cell (TIC) survival. Hypermethylation (epigenetic silencing) of Wnt-inhibitory factor 1 is a major hallmark of UCB. Additionally, upregulation of lncRNA UCA1 and Wnt 6 is associated with the development of chemoresistance [93].

Deregulated constitutive expression of the sonic hedgehog signaling cascade (Shh) is another key regulatory pathway associated with the development and progression of UCB. Constitutive activation of Shh and the subsequent decrease in the e-cadherin: N-cadherin ratio regulate EMT, invasion, and stemness. GANT1 facilitates Shh activation, enhancing self-renewal and increasing the tumorigenicity of UCB CSCs [94]. Apart from the activation of TGF-β, the PI3K/AKT, YAP1/COX2, and JAK/STAT signaling pathways increase features of stemness in UCB [21].

Research reports from our group have detailed CD24 as a key regulatory molecule responsible for cancer stem cell induction via modulating the activity of CD49f and NANOG. Overexpression of CD24 is reported not only in primary tumors, but also in patient urine samples [5]. Consequently, CD24 may serve as a promising molecule for noninvasive bladder cancer detection. Additional research from our lab supports YAP1 and COX2 as inducers of cancer stemness via SOX2 upregulation. In another project from our group, we report that exogenous exposure to arsenic leads to malignant transformation in normal urothelial cells (HUC-1) and induces cancer stemness by upregulating OCT4/NANOG via the COX2/PGE2-SOX2 pathway [5,21].

#### Evaluation of Targeted Therapy Activity against CSCs in Bladder Cancer

The current literature lacks thorough, detailed mechanistic research regarding FDA-approved targeted agents to reach bladder CSCs. In 2014, a comprehensive study by Kurtova et al. found that COX2/PGE2 signaling mediated the development of chemoresistance in CSCs [16]. A preclinical study showed that celecoxib treatment reduced the effect of PGE2 and the subsequent development of chemoresistance [21]. Upon further study, administering verteporfin (YAP1 inhibitor) and celecoxib (COX2 inhibitor)—two FDA-approved drugs for other pathological conditions—concurrently with systemic chemotherapy improved therapeutic efficacy in both cell-line-derived and patient-derived xenograft (PDX) models of UCB (17). In 2018, Shi et al. reported that a streptavidin-containing cancer vaccine exerted efficacy against urothelial CSCs. Furthermore, combination therapy with a PD-1 inhibitor induced T-cell differentiation (CD4+ and CD8+) [95]. This study reveals promise for UCB treatment and may serve as a template for future study design. At the time of this review, however, the efficacy of avelumab, durvalumab, and erdafitinib to inhibit CSCs has yet to be studied. Examination of these targeted drugs certainly warrants further investigation.

## 4. Conclusions

Therapy targeting CSCs as well as CSC-generating and -expanding pathways may yield promise for advances in cancer treatment; however, at present, the literature lacks sufficient evidence regarding targeted agents to reach these tumor cells. Overwhelmingly, preclinical and clinical data reveal the heterogeneity of CSC populations within and across cancer types. Prolonged exposure to chemotherapy without CSC-directed agents may compromise therapeutic efficacy as tumor features change and chemoresistance emerges. Despite advancements of knowledge in the repurposing of certain FDA-approved drugs [96,97], due to the highly heterogeneous nature and plasticity of CSCs, scientists are still in search of novel drug molecules for the effective inhibition of CSCs to overcome therapeutic resistance against chemotherapy and immunotherapy. Therefore, a detailed understanding of the molecular underpinnings and signaling crosstalk among CSCs is critical for effective treatment. In the era of personalized medicine, a broad foundation of in vitro and in vivo studies across cancer types is necessary to enhance selective tumor killing, inform clinical practice guidelines and, ultimately, improve patient outcomes.

## Figures and Tables

**Table 1:** List of common CSC markers in lung (LC), head and neck (HNC), and bladder cancers (BC).

Intracellular Factors	Common CSC Markers	LC	HNC	BC
Cell Surface Receptors	CD133	[21]	[39]	[52]
	CD44	[19]	[34]	[53]
	ALDH1A	[19]	[49]	[54]
Transcription Factors	OCT4	[19]	[41]	[54]
	BMI1	[19]	[42]	[54]
	SOX2	[5]	[19]	[54]
	NANOG	[39]	[54]	[5]

**Table 2:** List of potential CSC markers in lung, head and neck and bladder cancers.

Cancer Type	Potential CSC Markers	References
**Lung Cancer**		
Cell Surface Receptors	CXCR4	[32]
	CD90	[35]
	CD166	[36]
	uPAR	[37]
CSC Molecules	CEA	[39]
	EpCAM	
	NCAM	
Growth Factor	IGF-1	[27]
Membrane Transporter	ABCB1	[40]
	ABCG2	[55]
**Head and Neck Cancer**		
Cell Surface Receptors	CD166	[54]
	CD98	[54]
	CD117	[56]
**Bladder Cancer**		
Cell Surface Receptors	AR (Androgen receptor)	[19]
	CD24	[5]
	CD90	[21]
Transcription Factors	SOX4	[21]
Membrane Transporter	ABCG2	[21]
CSC-Related Proteins, Molecules	Nestin	[21]
	CK14	[21]
	CK5	[21]
	P-cadherin	[21]
	COX2/PGE2	[21]
	YAP1/STAT3	[24]
	Wnt/β-catenin	[19]

**Table 3:** List of FDA-approved agents demonstrating CSC-directed activity. The third column provides a brief description of the proposed mechanisms of the respective drug to reach CSCs.

FDA Approved Drugs	Pathway Blocked	Evidence of Generation and Expansion of CSCs
**Lung**		
Afatinib	EGFR	EGFR inhibition via NOTCH enriches cancer stem cell populations. Suggestion that combination therapy with STAT3 and Src inhibitors may improve blockade of CSCs [48,50]. In vivo, afatinib’s blockade of EGFR significantly suppresses PD-L1 expression via the inhibition of STAT1 and IRF1 [57].
Afinitor (Everolimus)	mTOR	Afinitor can deregulate the PI3K/AKT/mTOR pathway and dampen the CSC phenotype in the cancer cells. Clinical data also indicate its potential anticancer effects with paclitaxel in lung cancer patients.
Alectinib	ALK	Alectinib is an FDA-approved drug for ALK-positive lung cancer patients[58] showed its significant effect in combination with YAP1 inhibitors.
Bevacizumab	VEGF-A	Bevacizumab is approved by the FDA for the treatment of common primary brain tumors, and can prevent lung metastasis; it can attenuate tumor cell proliferation, and is simultaneously effective in relieving underlying disease symptoms.
Brigatinib	ALK, ROS1, IGF1, EGFR	Brigatinib is approved by the FDA for the treatment of metastatic non-small-cell lung cancer; it is capable of overcoming resistance against other ALK inhibitors.
Ceritinib	ALK	Ceritinib is approved by the FDA for lung cancer patients who have not been previously treated with ALK inhibitors; it also can dampen the CSC phenotype via the downregulation of PI3K-driven proliferative cascade.
Crizotinib	ALK, ROS1	Expression of linc-ROR and crizotinib concentration are negatively correlated. Linc-ROR elevates the viability of EML–ALK^+^ NSCLC cells, while crizotinib suppresses cell viability and CSC features. Thus, linc-ROR is a potential target for therapy with crizotinib [59]. Crizotinib-treated cells show decreases in NANOG, OCT4, and ALDH+ expression (all dose-dependent). Cells treated with crizotinib gradually and consistently lose the ability to form spheres, in a dose-dependent manner. Thus, the antitumor effect of crizotinib is at least partially related to the loss of stemness in these NSCLC cells [60].
Dabrafenib	BRAF	Dabrafenib has been shown to be effective against BRAF V600E-mutant non-small-cell lung cancer. The FDA has approved combinatorial use of this with trametinib for metastatic NSCLC patients. Dabrafenib mainly affects the CSC phenotype and cell proliferation by downregulating the MAPK cascade.
Dacomitinib	EGFR	Dacomitinib is effective against both EGFR-resistant and -sensitive advanced NSCLC patients.
Durvalumab	PD-L1	Durvalumab can effectively bind with PD-L1, but not PD-L2; it shows poor response against EGFR- and ALK-positive patients.
Erlotinib	EGFR	In EGFR wild-type cells, increased erlotinib activity was observed for cells with tyr1068 phosphorylation [61] Since EGFR wild-type CSCs express EGFR, the presence of tyr1068 may indicate the possibility of erlotinib response in CSCs [61]. Continuous exposure to erlotinib is associated with increased CSC trait expression, including CSC behaviors (self-renewal) and CSC molecules (NANOG, Oct 4, etc.). Treatment with anti-CSC molecules in combination with EGFR inhibitors may reduce the efficacy of these resistance mechanisms [31]. Erlotinib did not suppress the phosphorylation of PI3K and AKT in CSCs (despite EGFR inhibition). Compared to non-CSCs, there was an observed upregulation of miR-23a and downregulation of PTEN in CSCs. Knockdown of miR-23a may act as a mechanism to enhance the antitumor effect of erlotinib and increase subsequent apoptosis—novel strategy to eliminate the erlotinib resistance of lung cancer stem cells [62]. Erlotinib significantly reduces MDM2 levels. MDM2 is an oncoprotein that regulates p53 by inhibiting its transcriptional activity. However, there was no information on CSC markers [63]. Knockdown of SALL4 increased erlotinib sensitivity and promoted erlotinib-induced apoptosis. SALL4 knockdown reduced spheroid formation in vitro, as well as spheroid formation in CD44+ cells (CD44 is a surface marker of CSCs) [51]. Erlotinib-resistant NSCLC cells express markers of CSCs (CD44+, CD24-), and are able to form spheres more efficiently [31]. Expression of ABCG2 and CD133 was significantly elevated following treatment with erlotinib. Upon knockdown of MAP17, sphere cells were less resistant to erlotinib. Moreover, CSC cells with MAP17 knockdown decreased the efficiency of sphere formation [64]. Erlotinib-resistant cells had increased p120-catenin and Kaiso factor levels, which led to upregulation of EMT transcription factors. Via knockdown of p120-catenin and PRMT-1, cells were re-sensitized [28]. Erlotinib increased pSTAT3 and ALDH activity in EGFR mutant cells [48]. Silencing of DUOX1 led to enhanced resistance to erlotinib and upregulated levels of the CSC markers CD133 and ALDH1. The loss of DUOX1 was associated with acquired resistance to erlotinib and enhanced EMT and CSC features [65]. In lung CSCs, downregulation of miR-223 has been implicated in erlotinib resistance. Inhibition of miR-233 was observed in stem-like cells, and led to increased expression of IGF1R. The downregulation of miR-223 induces activation of the PI3k/AKT pathway in lung CSCs, and may also be responsible for the resistance of stem-like cells [66]. Elevated PGRMC1 levels were seen in lung tumor CSCs. However, no increased cancer stem cell death was noted with PGRMC1 inhibitor (AG-205) treatment as opposed to erlotinib treatment [67]. When coupled with Hedgehog pathway inhibitors, cells treated with erlotinib or gefitinib had decreased sphere formation [68].
Gefitinib	EGFR	EGFR inhibition through NOTCH enriches cancer stem cell populations. Cells that survived EGFR inhibition have elevated CSC marker expression. Moreover, single blockade of EGFR increases the population of CSCs. It has been suggested that combination therapy with STAT3 and Src inhibitors may improve blockade of CSCs [48]. Levels of FOXO3a (transcription factor that triggers apoptosis) are correlated with sensitivity to EGFR-TKI (gefitinib, erlotinib). Suppression of FOXO3a increases gefitinib resistance and enhances the stem-like properties of lung cancer cells. Moreover, miR-155 transcriptionally regulates NF-kB, leading to repressed FOXO3a, increased gefitinib resistance, and enhanced cancer stemness in vitro and in vivo [45]. Gefitinib acts upon CSC regulator SALL4 in CD44+ CSCs. SALL4 is associated with increased CSC characteristics, and is a potential target whose inhibition could decrease Gefitinib resistance [51].
Imatinib	SCF-c-kit	Imatinib inhibits c-kit, or CD177, signaling. The c-kit pathway is implicated in lung cancer CSC characteristics. Coupling with chemotherapy may inhibit CSC and bulk tumor cell growth [29].
Osimertinib	EGFR	EGFR inhibition through NOTCH enriches cancer stem cell populations. Cells that survive EGFR inhibition have elevated CSC marker expression. Single blockade of EGFR increases the population of CSCs. It has been suggested that combination therapy with STAT3 and Src may improve blockade of CSCs [48].
**Head and Neck**		
Cetuximab	EGFR	Cetuximab is ineffective in the CSC subpopulation of head and neck cancer cell lines, likely due to migratory behavior from EGFR expression [56]. Cetuximab also failed to consistently inhibit migratory behavior through different cell lines [69]. Cetuximab inhibits sphere formation [69] and decreases CD44 expression [70]. This is indicative of some chemical effects on CSCs in head and neck cancers.
Afatinib	EGFR, PD-L1	Afatinib can reduce SP cell count and CSC characteristics and increase CSC chemotherapy sensitivity via the inhibition of ABCG2 activity and expression. Activity on SP cells is likely independent of its EGFR activity. Thus, coupling with chemotherapy could lead to effective growth [71].
Erlotinib	EGFR	Erlotinib treatment is correlated with elevated CD133 and ABCG2 expression, possibly due to proliferation of TKI-resistant CSCs [67]. ALD (aldehyde dehydrogenase) enables CSCs to metabolize chemotherapy and oxidants. ALD can be targeted with the use of erlotinib in head and neck cancers [72].
